# Experimental fault-tolerant universal quantum gates with solid-state spins under ambient conditions

**DOI:** 10.1038/ncomms9748

**Published:** 2015-11-25

**Authors:** Xing Rong, Jianpei Geng, Fazhan Shi, Ying Liu, Kebiao Xu, Wenchao Ma, Fei Kong, Zhen Jiang, Yang Wu, Jiangfeng Du

**Affiliations:** 1 Hefei National Laboratory for Physical Sciences at the Microscale, University of Science and Technology of China, Hefei 230026, China; 2 Department of Modern Physics, University of Science and Technology of China, Hefei 230026, China; 3 Synergetic Innovation Center of Quantum Information and Quantum Physics, University of Science and Technology of China, Hefei 230026, China

## Abstract

Quantum computation provides great speedup over its classical counterpart for certain problems. One of the key challenges for quantum computation is to realize precise control of the quantum system in the presence of noise. Control of the spin-qubits in solids with the accuracy required by fault-tolerant quantum computation under ambient conditions remains elusive. Here, we quantitatively characterize the source of noise during quantum gate operation and demonstrate strategies to suppress the effect of these. A universal set of logic gates in a nitrogen-vacancy centre in diamond are reported with an average single-qubit gate fidelity of 0.999952 and two-qubit gate fidelity of 0.992. These high control fidelities have been achieved at room temperature in naturally abundant ^13^C diamond via composite pulses and an optimized control method.

## Abstract (web summary)

High fidelity manipulation of diamond-based spin qubits is difficult at room temperature because of decoherence. Here, the authors show a universal set of logic gates in nitrogen-vacancy centres with average single-qubit and two-qubit gate fidelities of 0.999952 and 0.992, respectively.

## Graphical abstract

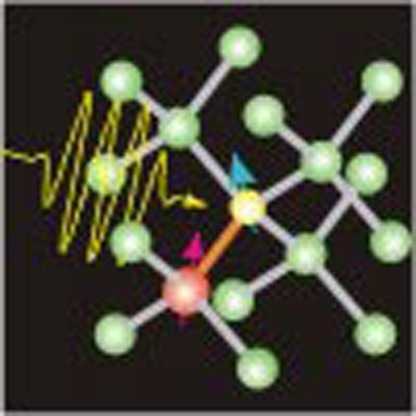

Quantum computations promise solutions of certain intractable problems in classical computations, such as quantum simulations^1^,^2^, prime factoring^3^,^4^, and machine learning^5^,^6^. Recently, exciting progress towards spin-based quantum computation has been made with nitrogen-vacancy (NV) centres in diamond, which is a promising candidate for quantum computation under ambient conditions^7^. Long coherence time of NV centres has been achieved with dynamical decoupling technique^8^. Robust single-qubit and two-qubit gates have been accomplished^9^,^10^,^11^,^12^. NV–NV entanglement has been realized showing the scalability of this quantum system^13^,^14^. Quantum algorithms^15^,^11^ and quantum error correction^16^,^17^ have been recently reported in NV centres. Further improvement of NV centre towards realistic quantum computation would require high fidelity quantum gates with errors below fault-tolerant threshold, which is proposed to be between 10^−4^ and 10^−2^ depending on the noise model and the computational overhead for realizing quantum gates^18^,^19^,^20^. Although fault-tolerant control fidelity has been reported very recently in superconducting qubits^21^, trapped ions^22^ and phosphorus doped in silicons^23^, it is still of great challenge to achieve fault-tolerant fidelity under ambient conditions, which is the case for NV centre in ^13^C naturally abundant diamond at room temperature. It is because the noise, not only stemming from interactions between the quantum system and the environment but also induced by imperfect manipulations, limits the fidelities of quantum gates.

In the following, a universal set of high-fidelity quantum gates at the threshold for fault-tolerant quantum computations in the NV centre system are realized. A novel composite pulse technique has been developed to suppress the noises during the single-qubit gates. We adopt the quantum optimal control method for designing the two-qubit gate to suppress the effects induced by both the spin bath and the imperfect control field. A novelly designed coplanar waveguide (CPW) with 15 GHz bandwidth has been fabricated to minimize the effect owing to the finite bandwidth for the microwave pulses. Pulse-fixing technique is utilized to correct the effect of the imperfect generation of the microwave pulses. The average gate fidelity of single qubit is measured to be 0.999952(6) and the fidelity of the two-qubit controlled-NOT (CNOT) gate reaches 0.9920(1). Thus, we have successfully demonstrated a universal set of quantum gates with the fault-tolerant control fidelity in diamonds.

## Results

### NV centre in diamond

**Figure 1:**
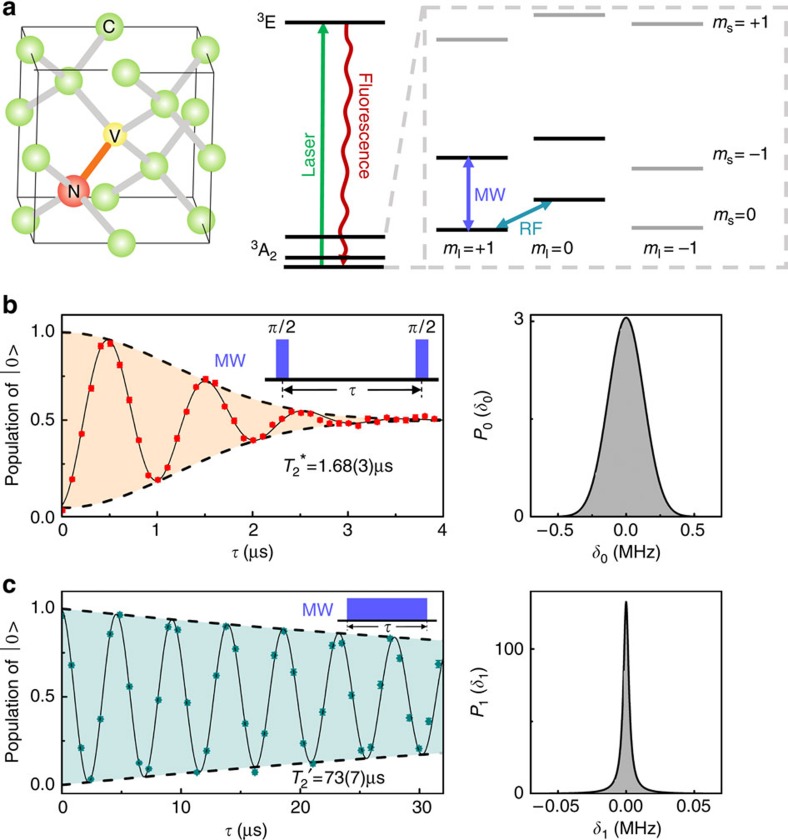
Characterization of the noises in experiment. (**a**) Schematic atomic and energy levels of the NV centre. Laser pulses are used for state initialization and readout. Microwave pulses (MW) are used for coherent control of the electron spin. Radiofrequency pulses (RF) are used for manipulating the nuclear spin. (**b**), Result of the Ramsey experiment (inset, pulse sequence) for the electron spin. The solid black line in the left panel is fit to the experimental data (red circles) with *P*_0_(*δ*_0_), which is the probability density distribution of *δ*_0_ depicted in the right panel. The decay time of FID is measured to be 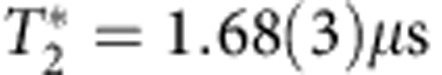. (**c**), Results of the nutation experiment (inset, pulse sequence) for the electron spin. The stepped MW pulse length is set to be 810 ns. The decay time of the nutation is 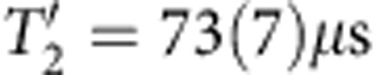. The solid black line in the left panel is fit to the experimental results (olive diamonds) with *P*_1_(*δ*_1_), the probability density distribution of *δ*_1_ depicted in the right panel. The error bars on the data points are the standard deviations from the mean.

Figure 1a depicts the NV centre in diamond and the energy level structure. The NV centre consists of a substitutional nitrogen atom with an adjacent vacancy site (V) in the diamond crystal lattice. The ground state of NV centre is an electron spin triplet state ^3^A_2_, with three sublevels 
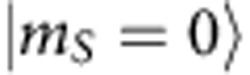
 and 
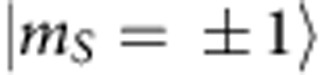
. The intrinsic nitrogen nuclear spin results in further splitting of the energy levels owing to the hyperfine coupling. In the experiment presented here, the two-qubit system comprises the electron spin qubit and ^14^N nuclear spin qubit. Electron (nuclear) spin states 
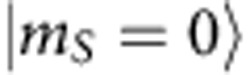
 and 
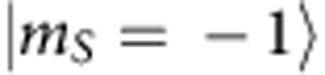
 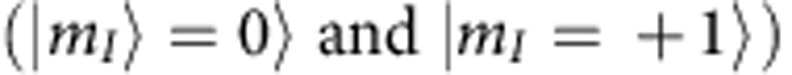 are encoded as the electron (nuclear) spin qubit (Fig. 1a). When a 532 nm green laser pulse is applied to the NV centre, the electron spin will be excited to the ^3^*E* state, and then fluorescence emission can be measured. The optical transitions are used to initialize and read out the state of the electron spin. The polarization of the electron spin is measured to be 0.83(2) (see Supplementary Note 8 and Supplementary Fig. 9). A magnetic field of 513 Gauss is applied along the NV symmetry axis ([111] crystal axis) to split the 
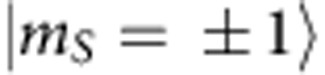
 energy levels, and to polarize the nitrogen nuclear spin (Supplementary Note 5).

### Realization of high-fidelity single-qubit gates

The high-fidelity single-qubit gate is demonstrated on the electron spin qubit. The state evolution of the electron spin qubit is governed by the Hamiltonian 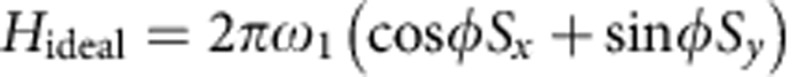 in the rotating frame, where *φ* is the microwave phase and *ω*_1_ is the Rabi frequency. Unfortunately, this is only the ideal case. In practice, the Hamiltonian is 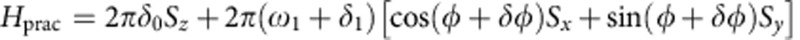, where *δ*_0_, *δ*_1_ and *δφ* lead to gate error. The *δ*_0_ mainly comes from the Overhauser field (due to the interaction with the nuclear spin bath), the magnetic field fluctuation and the instability of the microwave frequency. The *δ*_1_ partly comes from the control field error owing to the static fluctuation of the microwave power. The distortion of the pulse owing to the finite bandwidth also contributes to the *δ*_1_. The *δφ* describes the distortion of the microwave phase owing to the imperfect microwave generation. We take the distortion of the amplitude and the phase of the microwave pulse as the systematic errors, which have unchanged deviations from the ideal cases during each experimental scan. The other errors can be treated as the fluctuation of the experimental parameters. All of these errors limit the fidelity of quantum gates.

We first quantitatively characterize the distributions of errors owing to the fluctuation of the experimental parameters. As the timescale of variation of *δ*_0_ and *δ*_1_ is much longer than that of the quantum gates, *δ*_0_ and *δ*_1_ can be taken as quasi-static random constants, and the practical Hamiltonian is rewritten as 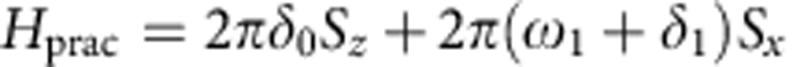. The fluctuations of *δ*_0_ and *δ*_1_ lead to the decoherence of the qubit during quantum gates when averaged over repeated experiments. Figure 1b,c show the experimental distributions of *δ*_0_ and *δ*_1_ derived by free induction decay (FID) and nutation experiments, respectively. The distribution of *δ*_0_, that is, *P*_0_(*δ*_0_), is obtained from the FID signal via the Ramsey experiment, which is shown in the left panel of Fig. 1b. The oscillation of the FID signal is owing to the detuning of the microwave frequency. We assume that *δ*_0_ satisfies a Gaussian distribution 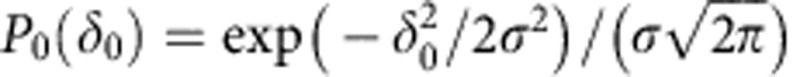, where *σ* stands for the standard deviation of the distribution. The fitting of the FID data is based on distribution *P*_0_(δ_0_), with parameter *σ* optimized to achieve best agreement between the fitted and experimental data. The fitted data with best agreement are shown as the solid curve in Fig. 1b, which gives *σ*=0.131(5) MHz, with the probability density distribution *P*_0_(*δ*_0_) depicted in the right panel of Fig. 1b. The distribution of *δ*_1_, that is, *P*_1_(*δ*_1_), obtained via nutation experiments, is shown in Fig. 1c. The Rabi frequency is set to be *ω*_1_=10 MHz. The *δ*_1_ satisfies a Lorentzian distribution of 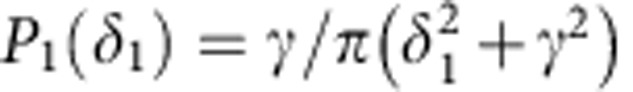, where *γ* is the half-width at half-maximum of the distribution. The distribution *P*_1_(*δ*_1_) together with *P*_0_(*δ*_0_) obtained from the FID, is used to fit the nutation experiment. Best agreement between the fitted and experimental data is achieved with *γ*=0.0024(4) MHz. The fitting result is the solid curve in the left panel of Fig. 1c. The probability density distribution *P*_1_(*δ*_1_) is depicted in the right panel of Fig. 1c.

**Figure 2:**
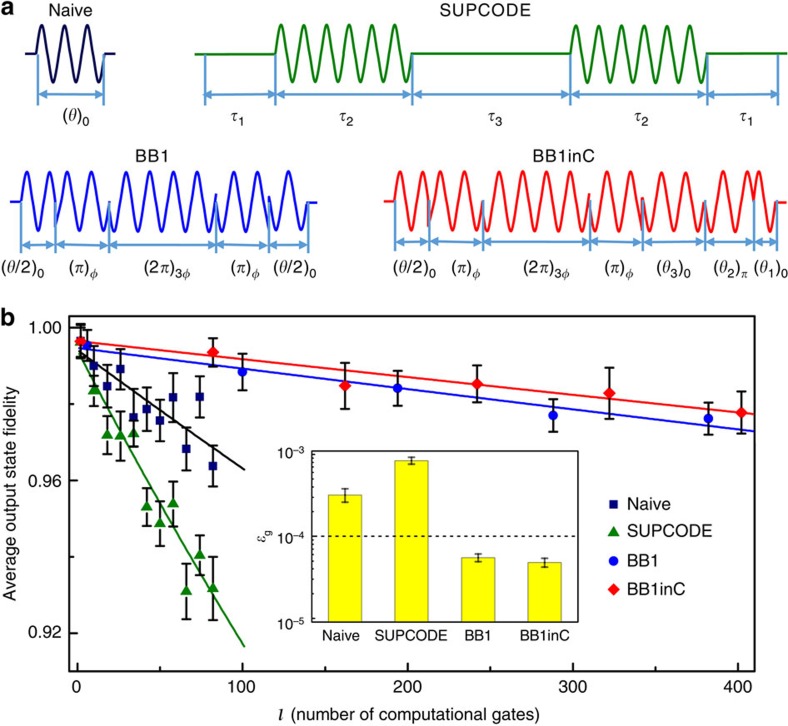
Characterization of the performance of single-qubit gates. (**a**) Pulse sequences corresponding to naive pulse, SUPCODE pulse, BB1 pulse and BB1inC pulse. The time durations *τ*_*i*_ (*i*=1, 2 and 3) of SUPCODE is included in Supplementary Note 3. (**b**) Results of randomized benchmarking. Red diamonds, blue circles, navy blue squares and green triangles represent measured single-qubit fidelity of the output state from each of the individual sequences of gates. Solid lines are fits of equation (2) with averaging each over all the randomization pulse sequences. The average gate fidelity for the naive, five-piece SUPCODE, BB1 and BB1inC pulses are 0.99968(6), 0.99916(8), 0.999945(6) and 0.999952(6) respectively. The inset shows the average error per gates (ɛ_g_) of the pulses. The error bars on the data points are the standard deviations from the mean, and those of ɛ_g_ in inset are given by errors of the fit of the randomized benchmarking data.

Since distributions of *δ*_0_ and *δ*_0_ have been quantitatively characterized, we now demonstrate the realization of high-fidelity gates with a novel composite pulse, which is designed to suppress these noises simultaneously. To form the new composite pulse, we replace one of the constituent elementary pulses of CORPSE (compensation for off-resonance with a pulse sequence) by BB1 (broadband number 1) sequence. This new composite pulse is named as BB1inC for short. Theoretical calculation shows that BB1inC is robust against both errors of *δ*_0_ and *δ*_1_ (Supplementary Note 3 and Supplementary Fig. 1). We experimentally compare the performance of this new pulse sequence with three other types of pulse sequences, which are naive (rectangular) pulse, SUPCODE (soft uniaxial positive control for orthogonal drift error)^24^ pulse, and BB1 pulse^25^. The rotation of an angle *θ* around the axis in the equatorial plane with azimuth *φ* is denoted by (*θ*)_*φ*_. The naive pulse is very sensitive to the errors *δ*_0_ and *δ*_1_, with leading orders of both errors preserved in the evolution operator (corresponding to second orders in gate fidelity). The SUPDODE pulse, a type of dynamically corrected gate, has been proposed to suppress the dephasing noise during quantum gates. The five-piece SUPCODE pulse has been used here, where the waiting time *τ*_1(3)_ and the pulse duration *τ*_2_ satisfy the specific requirement (see Supplementary Note 3). Under the five-piece SUPCODE pulse, up to second order of *δ*_0_ can be canceled (corresponding to sixth order preserved in gate fidelity). This has recently been demonstrated in NV centres^10^ with control field *ω*_1_ of 1 MHz. However, if we increase the control field to shorten the gate time, the fluctuation of the control field will dominate the decrease of the gate fidelity. A composite pulse, named BB1, which is shown to be robust against the *δ*_1_, can be applied to suppress the fluctuation of the control field. This pulse sequence is 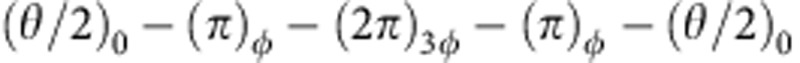, with 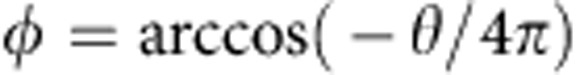. The error induced by *δ*_1_ is inhibited up to second order in the evolution operator. The sequence of BB1inC, which consists of seven pulses, is shown in Fig. 2a. The BB1inC sequence is depicted as 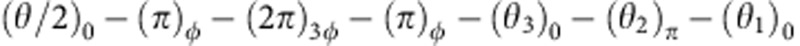, where 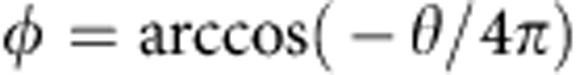, 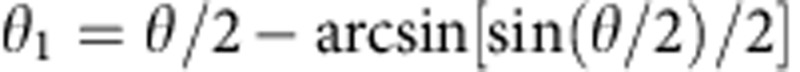, 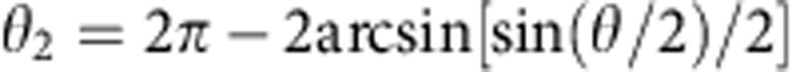, and 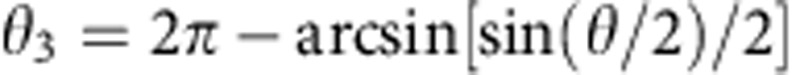. Leading orders of both the *δ*_0_ and *δ*_1_ errors are canceled in the evolution operator, so BB1inC can suppress both errors simultaneously.

The average gate fidelity^26^ between a quantum operation *ξ* and a target unitary quantum gate *U* is defined as


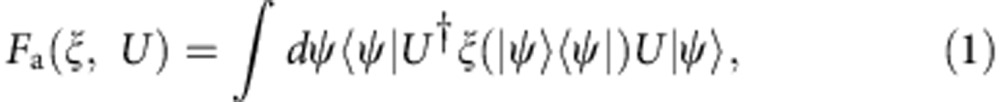


where the integral is over the uniform measure 
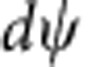
 on the Hilbert space of the system. To estimate the fidelity by equation (1), it is necessary to characterize the quantum operation *ξ* with comprehensive knowledge about the Hamiltonian of the quantum system, and the Hamiltonian of the control together with the errors during the gate operation. We numerically calculate the gate fidelities (Supplementary Note 2) for these pulse sequences with both *δ*_0_ and *δ*_1_. The BB1inC pulse presents a desirable region with fidelity higher than 0.9999 (Supplementary Note 3 and Supplementary Fig. 1). The control field strength is set to *ω*_1_=10 MHz for experimental realization of high-fidelity single-qubit gates, so that the error due to *δ*_0_ and *δ*_1_ can be greatly reduced.

Though composite pulses provide possibility to realize high-fidelity gates in solid-state system, the performance of the composite pulses is limited by the systematic errors, which are usually not taken into account in the composite pulse designing. Herein, we present methods to correct the systematic errors. The distortion of the microwave phase is corrected by pulse-fixing technique, which is previously developed in liquid NMR (nuclear magnetic resonance)^27^. The phase of the microwave pulses was recorded by an oscilloscope, and then the deviation of the phase from the ideal case was fed back to the arbitrary waveform generator so that this distortion can be minimized (see Supplementary Note 6 and Supplementary Fig. 5). The distortion of the amplitude is primarily owing to the limited bandwidth of the microwave-fed structure. We designed and fabricated an ultra-broadband CPW with a bandwidth up to 15 GHz (see Supplementary Note 5 and Supplementary Fig. 4). Then the effect of the finite bandwidth is found to be negligible. The reflection between the microwave components also contributes to the imperfection of the microwave pulses. This effect was diminished by inserting proper attenuators between the microwave components (Supplementary Note 6 and Supplementary Fig. 6). The amplitude distortion is further corrected with pulse fixing (Supplementary Note 6 and Supplementary Fig. 7). After all these procedures, the microwave pulses can be fed to the electron spin with almost perfect pulse shapes. The detailed procedures for correcting the systematic errors are included in the Supplementary Note 6.

To experimentally quantify the performance of the single-qubit gates, we utilize the randomized benchmarking (RB) method^28^. In the RB experiment, quantum gates are evaluated by measuring the performance when random sequences of the gates are applied. The error owing to the imperfect measurement and state preparation can be separated, and the gate fidelity can be determined precisely. When the number of randomized gates implemented in a sequence is increased, the accumulated gate error reduces the measured single-qubit fidelity *F* of the output state, which is defined as the overlap of the ideal and the measured states. The average output state fidelity 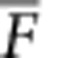 (ref. 28) can be written as


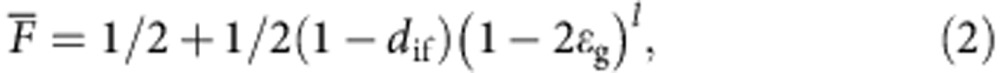


where *d*_if_ is owing to the imperfection of the state initialization and readout, *ɛ*_g_ is the average error per gate and *l* is the number of randomized quantum gates. The average gate fidelity 
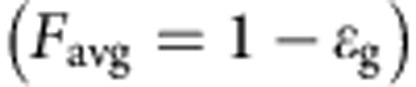
 derived from this method is resilient to the state preparation and measurement errors. The detailed procedure of RB is included in the Supplementary Note 9. Figure 2b shows the result of the randomized benchmarking, where the control field *ω*_1_ is set to be 10 MHz. The average gate fidelity of naive pulses is found to be 0.99968(6). The five-piece SUPCODE pulses provide an average gate fidelity of 0.99916(8), which is lower than that of the naive one. This is because the fluctuation of control field dominates the decay of the fidelity when *ω*_1_=10 MHz. The average gate fidelity is greatly improved when BB1 pulses are applied. The extracted average gate fidelity for BB1 pulses is 0.999945(6). The highest fidelity is achieved when BB1inC pulses are used. The average gate fidelity for BB1inC is 0.999952(6), providing an error per gate below 10^−4^ which is about one order of magnitude lower than that of naive pulses (Supplementary Table 1).

### Realization of high-fidelity CNOT gate

Two-qubit CNOT gate together with the single-qubit gates provide a universal set of quantum gates. Two-qubit gates have been demonstrated in NV centres^11^,^12^. However, realization of the two-qubit gates with high fidelity to meet the requirement of the fault-tolerant quantum computation is still of great challenge. The control qubit is the nuclear spin qubit and the target qubit is the electron spin qubit. The detailed Hamiltonian is included in Supplementary Note 1. The CNOT gate is designed to flip the electron spin qubit if the nuclear spin qubit is in the state 
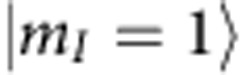
. Microwave and radio-frequency (RF) pulses are used to manipulate this two-qubit system.

**Figure 3:**
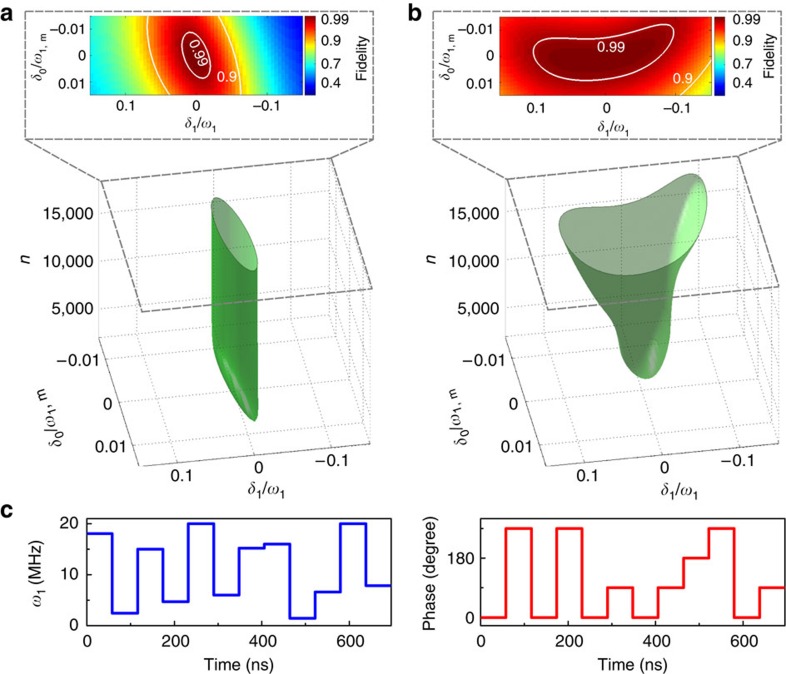
Optimization of the control of the two-qubit system to achieve high fidelity control in the presence of noises. (**a**,**b**) Comparison of the original GRAPE (**a**) with the modified one (**b**) in the presence of the noises *δ*_0_ and *δ*_1_. Here *ω*_1, m_ stands for the maximal strength of the control field in the pulse sequence and *n* stands for the number of iterations in the algorithms. The upper figures of **a**,**b** are the fidelity contour maps of the two GRAPE methods. The area with a control fidelity higher than 0.99 is larger for the modified method than the original one. (**c**) The schematic diagram of the optimal control pulses used in the experiment, with the left and right being the amplitude and phase sequences, respectively.

To achieve high fidelity, we have improved a type of quantum optimal control method, named GRAPE (gradient ascent pulse engineering)^29^, to design the CNOT gate which is robust against both *δ*_0_ and *δ*_1_. GRAPE has recently been used to realize the quantum error correction^16^ and a high-fidelity entanglement^13^ in diamond. Because the nuclear spin is insensitive to the noise (such as fluctuations of the external magnetic field and the control RF field) and the CNOT gate designed by the quantum optimal control method consists of microwave pulses only, we can take into account the noise felt by the electron spin in the optimization procedures. We modified the GRAPE algorithm to design target gates that are robust against both the errors *δ*_0_ and *δ*_1_ (see Supplementary Note 4 and Supplementary Fig. 2). Figure 3 shows the optimization of microwave pulse parameters to achieve the high control fidelity. Figure 3a,b shows the optimizing procedure of original (modified) GRAPE algorithm. The final achieved high-fidelity regions with the two GRAPE algorithms are compared in the upper parts of Fig. 3a,b. It is clear that the modified method presents much more robustness against both *δ*_1_ and *δ*_0_ than the original one. The left and right panels of Fig. 3c show the amplitude and phase sequences after the improved optimization, respectively. The total length of the sequence for one CNOT gate is 696 ns, which consists of 12 pulses of equal length with different amplitudes and phases. The theoretical fidelity of the CNOT gate via this pulse sequence is estimated to be 0.9995 in the absence of *δ*_0_ and *δ*_1_. If the noises *δ*_0_ and *δ*_1_ are taken into account, the average gate fidelity of the designed CNOT gate is estimated to be 0.9927. The detailed pulse optimization and fidelity estimation are included in the Supplementary Note 4.

**Figure 4:**
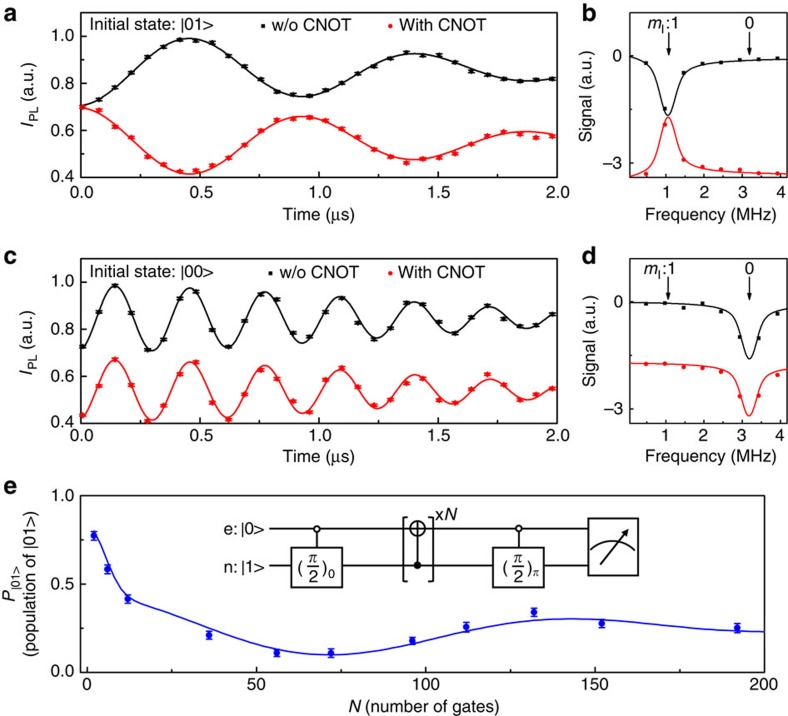
Characterization of the performance of CNOT gate. (**a**,**c**) These are the experimental data and the fittings of FID (black: without applying the CNOT gate, red: with applying the CNOT gate), when the initial states are 
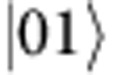
 and 
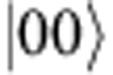
, respectively. Lines are fits to the experimental data (symbols with error bars). (**b**,**d**) These are the fast Fourier transform results of FID signals. (**e**) The probability in the state 
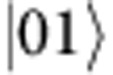
 
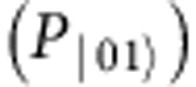
 after applying CNOT gates repeatedly, with the sequence shown in the inset. The fitting (blue line) agrees with the experimental data (blue circles with error bars). The error bars on the data points are the standard deviations from the mean. The detailed procedure of normalization to obtain 
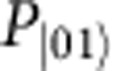
 is included in Supplementary Note 7 and Supplementary Fig. 8.

Figure 4 presents the experimental realization of the CNOT gate. The two-qubit system is prepared to the state 
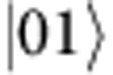
 and 
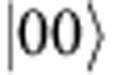
 in Fig. 4a,c, respectively (The state 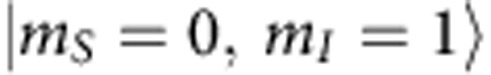 is denoted as 
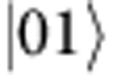
 and the state 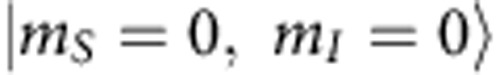 as 
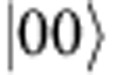
 hereafter). The black (red) symbols with error bars in Fig. 4a,c are the experimental FID signal via the Ramsey experiment without (with) the CNOT gate. Figure 4b,d are the fast Fourier transformation of the FID signals, so that the result of the CNOT gate can be observed in the frequency domain. The two peaks correspond to the nuclear spin qubit states 
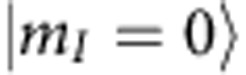
 and 
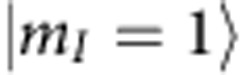
. The distance between the two peaks is due to the hyperfine coupling. It is clear that when the nuclear spin qubit is of state 
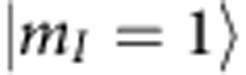
, the electron spin qubit is rotated by π (Fig. 4a,b). If the nuclear spin qubit is of state 
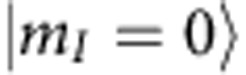
, the electron spin qubit remains unchanged (see Fig. 4c,d).

Two-qubit randomized benchmarking, which can be used to measure the fidelity of two-qubit gate, requires operations on both qubits^21^. In the hybrid system composed of electron and nuclear spins, single-qubit gates of the nuclear spin cost much longer time than that of the electron spin qubit. The typical operation time on the nuclear spin qubit (∼50 μs for a *π* rotation) is much longer than the dephasing time 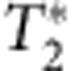 of electron spin qubit. The decoherence effect on the electron spin during the nuclear spin operation in the two-qubit RB experiments will dominate the fidelity decay in randomized benchmarking, and the gate fidelity of CNOT cannot be precisely determined in this way. On the other hand, though process tomography provides a full characterization of CNOT gate, the measured gate fidelity with this method is sensitive to errors in state preparation and measurement. In this scenario, repeated application of CNOT gates on the system and recording the dynamics of the quantum state^30^ will be a good choice to estimate the gate fidelity.

Figure 4e shows the population of 
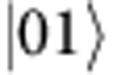
, 
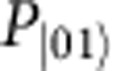
, after repeated applications of CNOT gates on the input state, which is generated by a selective *π*/2 RF pulse. The inset of Fig. 4e shows the pulse sequence, where the number of repeated CNOT, *N*, is even. When *N* is increased, the error of the CNOT gate will accumulate and 
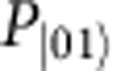
 will decay. A wealth of information can be obtained by studying the state dynamics under repeated applications of CNOT gates. In ref. 30, the dynamics of states obey a simple exponential decay and the gate fidelity can be extracted with 12 applications of CNOT gates. However, when more CNOT gates (up to 192 in this work) are applied, we find that the decay is not exponential. So the gate fidelity cannot be simply extracted from the decay as presented in ref. 30. In Fig. 4e, the dynamics of the state exhibits oscillatory attenuation as *N* increases. The oscillation results from the deviation of the realistic evolution from the ideal CNOT operation. For example, the optimization procedure adopts the hyperfine coupling *A*=−2.16 MHz, which may differ slightly from the experimental value *A*_exp_ by *δA*=*A*_exp_−*A*. The frequency of the microwave will not be equal to the resonant frequency exactly, which induces an off-resonance term (*δ*Ω) in the practical Hamiltonian. The decay of the 
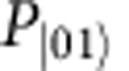
 is mainly owing to the static fluctuation of *δ*_1_ and *δ*_1_, which can be quantitatively characterized by the FID and the nutation experiment, respectively. The values of *δA* and *δ*Ω were derived to be 0.008 MHz and 0.068 MHz, by fitting the experimental data (Supplementary Note 9 and Supplementary Fig. 10). The fitting result shown as the solid blue line in Fig. 4e agrees with the experimental data. Since the comprehensive information on the Hamiltonian of the quantum system and the control field together with the errors is ready, the gate fidelity of CNOT can be directly obtained according to equation (1) with 0.9920(1) (see Supplementary Note 9).

## Discussion

To sum up, we have achieved fault-tolerant control fidelity for a universal set of quantum gates in diamond with ^13^C of natural abundance under ambient conditions. Several errors which limit the fidelity of quantum gates have been quantitatively characterized and effectively suppressed. With new composite pulses, we have realized single-qubit gate with fidelity up to 0.999952. A modified optimal control method has been developed to design the control pulse for the CNOT gate, which achieves a gate fidelity of 0.992. To the best of our knowledge, our results stand for the state-of-art high-fidelity control of solid-state spins under ambient conditions. Our method can not only be used to realize high fidelity CNOT gate in the system consisting of the electronic spin and the host nitrogen nuclear spin in NV, but also can be applied for the coupled NV–NV systems, which provide possibilities for future scalable architectures. The noises which limit the fidelity of the two-qubit gate in NV–NV system can also be greatly suppressed and high-fidelity gates are available by our method (Supplementary Note 10 and Supplementary Fig. 11). The methods presented here to achieve high control fidelity can also be applied to other quantum systems, such as quantum dots, phosphorus doped in silicon, and trapped ions.

## Methods

### Experiment setup

The NV centre in [100] face bulk diamond was mounted on a typical optically detected magnetic resonance confocal setup, which was synchronized with the microwave bridge by a multichannel pulse generator (Spincore, PBESR-PRO-500). The nitrogen concentration was less than 5 p.p.b. and the abundance of ^13^C was at the natural level of 1.1%. The 532-nm green laser for pumping and phonon sideband fluorescence (650–800 nm) went through the same oil objective (Olympus, PLAPON 60XO, NA 1.42). To preserve the NV centre's longitudinal relaxation time *T*_1_ from laser leakage effects, the pump beam was passed twice through an acousto–optic modulator (ISOMET, power leakage ratio ∼1/1,000) before it went into the objective. We created a solid immersion lens in the diamond around an NV centre (see Supplementary Note 5 and Supplementary Fig. 3). The solid immersion lens increases the PL rate to ∼400 kcounts s^−1^. The fluorescence intensity was collected by avalanche photodiodes (Perkin Elmer, SPCM-AQRH-14) with a counter card. The adjustable external magnetic field, created by the permanent magnets, was aligned by monitoring the variation of fluorescence counts. Microwave and radio frequency pulses were carried by ultra-broadband CPW with 15 GHz bandwidth.

## Additional information

**How to cite this article:** Rong, X. *et al*. Experimental fault-tolerant universal quantum gates with solid-state spins under ambient conditions. *Nat. Commun.* 6:8748 doi: 10.1038/ncomms9748 (2015).

## Supplementary Material

Supplementary Information Supplementary Figures 1-11, Supplementary Table 1, Supplementary Notes 1-10 and Supplementary References
