# Supplementary material for: Experimental fault-tolerant universal quantum gates with solid-state spins under ambient conditions
Source: Nat Commun. 2015 Nov 25;6:8748. doi: 10.1038/ncomms9748 (PMC4674779; doi:10.1038/ncomms9748)
Supplement: Supplementary Information — Supplementary Figures 1-11, Supplementary Table 1, Supplementary Notes 1-10 and Supplementary References [file ncomms9748-s1.pdf]

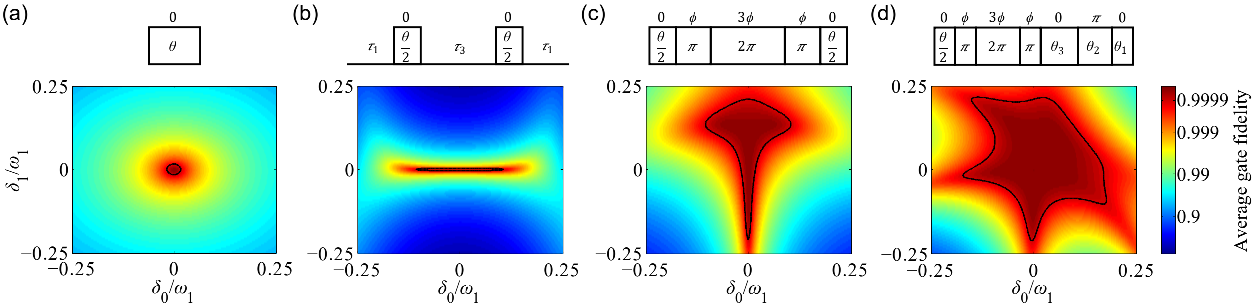

Supplementary Figure 1: **Pulse sequences and contour plot of average gate fidelity of single-qubit gate.** The upper panels show the pulse sequences of $(\theta)_0$ with (a) rectangular naive, (b) five-piece SUPCODE, (c) BB1, and (d) BB1inC pulses, where the phase and duration of each piece of pulse is depicted. The lower panels show the average gate fidelity of $(\pi/2)_0$ with the errors δ_0 and δ_1 when (a) rectangular naive, (b) five-piece SUPCODE, (c) BB1, and (d) BB1inC pulses are applied. The regions of fidelity larger than 0.9999 are surrounded with black curves for clarity.

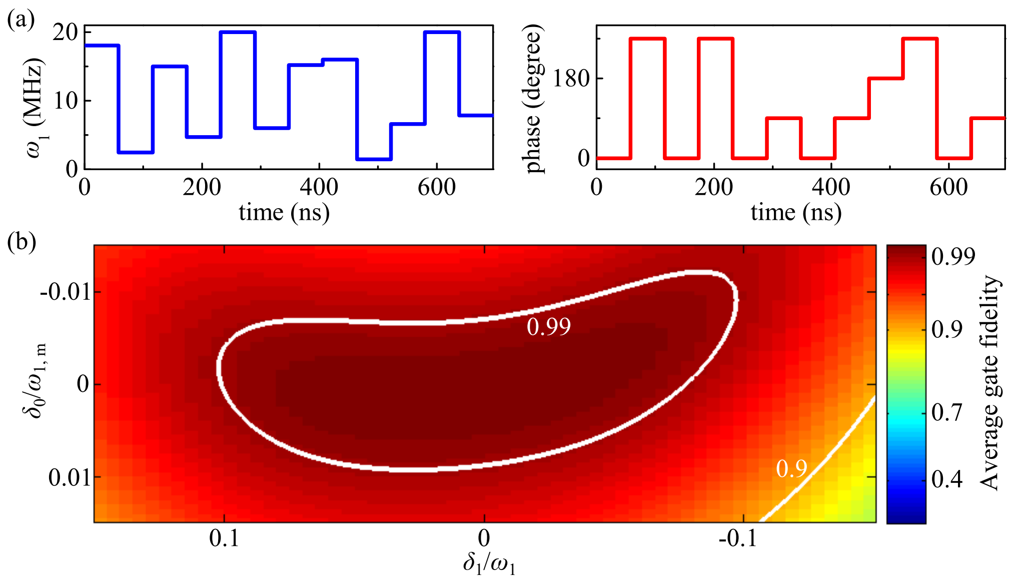

Supplementary Figure 2: **Designed pulse sequence for CNOT and average gate fidelity of the sequence.** (a) Amplitude (left panel) and phase (right panel) of the designed pulse sequence. (b) Calculated average gate fidelity of the sequence under the errors δ_0 and δ_1.

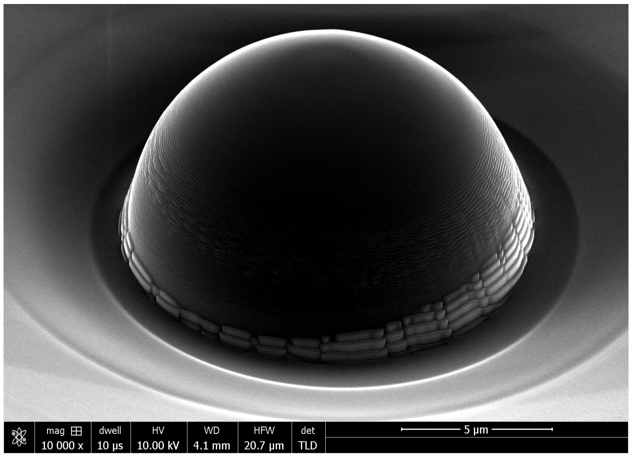

Supplementary Figure 3: **Image of the SIL in diamond.**

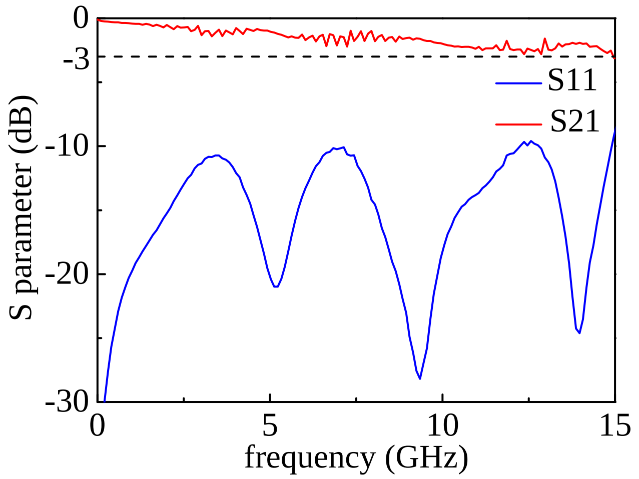

Supplementary Figure 4: **Scattering parameters of the CPW.**

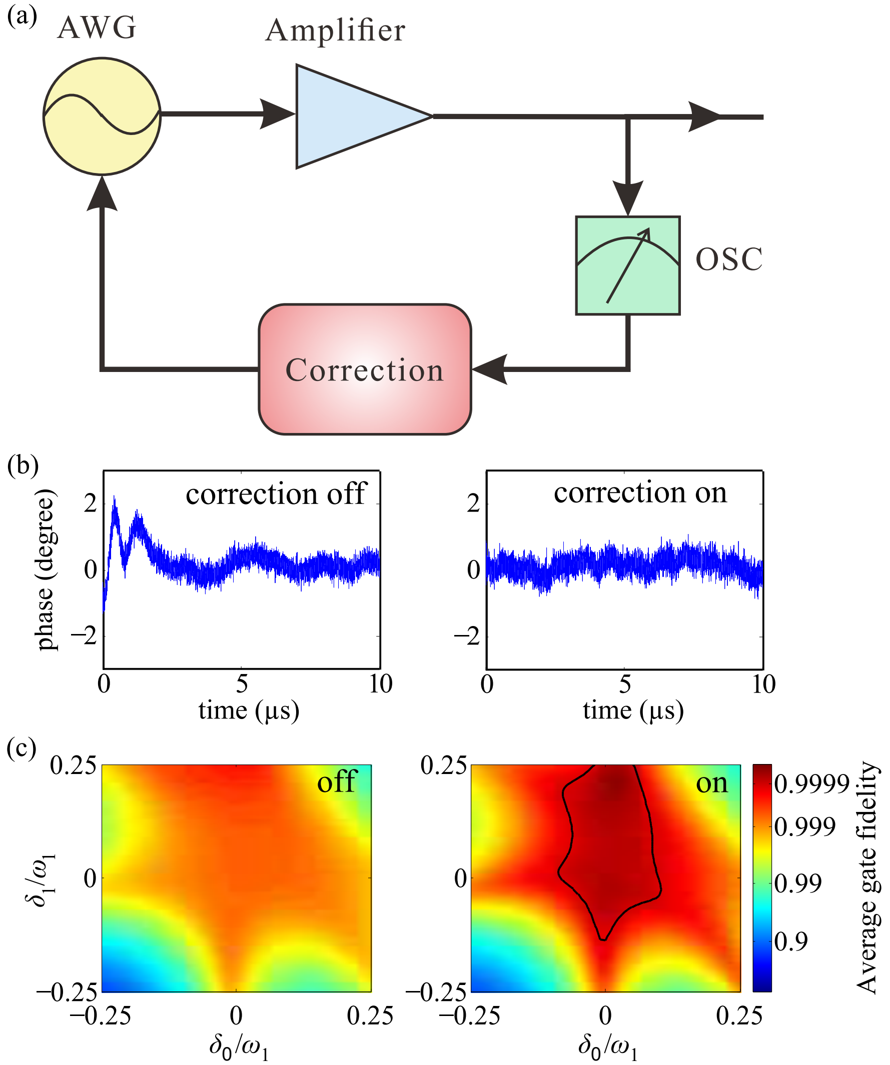

Supplementary Figure 5: **Correction of microwave pulse distortions.** (a) Schematic correction diagram showing the main instruments generating, processing, and sampling the microwave pulses. (b) Extracted phase distortions without (left penal) and with (right penal) the correction. (c) Calculated average gate fidelity of a BB1inC $\pi/2$ gate with sampled waveforms without (left penal) and with (right penal) the correction. The region of fidelity larger than 0.9999 is labeled.

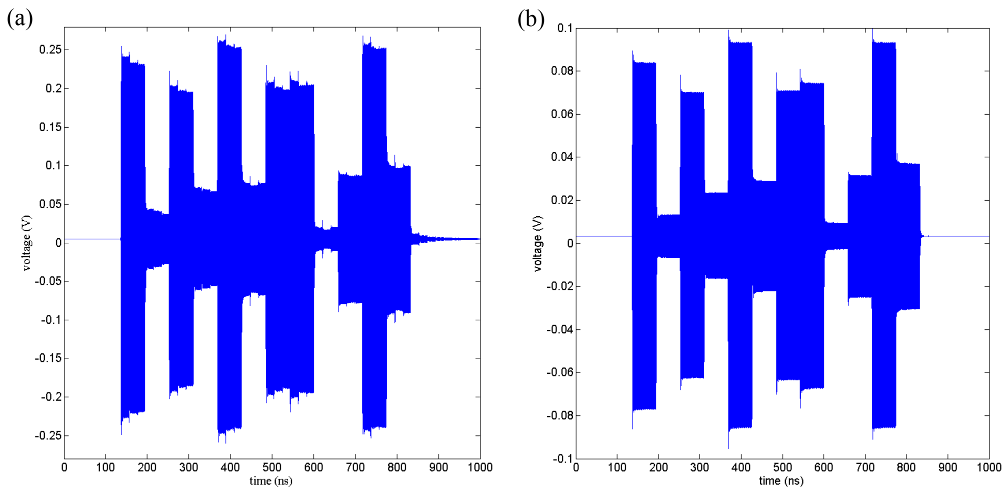

Supplementary Figure 6: **Correction of pulse distortions caused by leakage and reflection.** (a) Waveform of a GRAPE pulse without improvement. The distortions are mainly caused by leakage and reflection of the diplexer. (b) Improved waveform of the pulse sequence. The distortions are corrected by inserting a 10 dB attenuator between microwave components.

(a) (b)

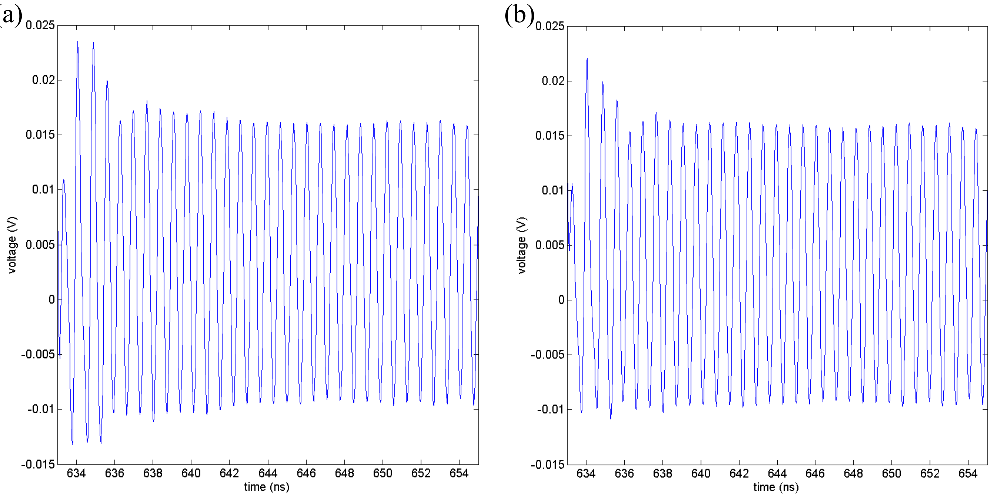

Supplementary Figure 7: **Correction of the amplitude distortions.** (a) Waveform without correction. (b) Waveform with correction.

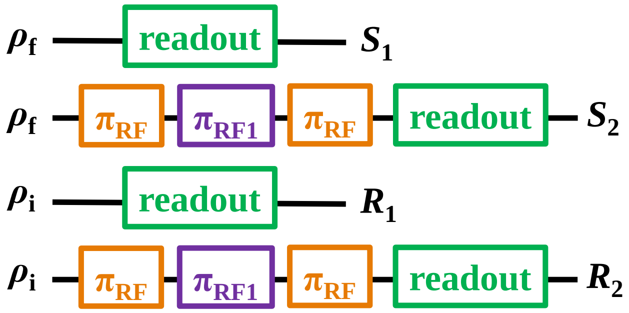

Supplementary Figure 8: **Schematic normalization sequences in the two-qubit experiment.** Here ρ_i denotes the initialized state with laser, ρ_f denotes the final state after applying control sequence to ρ_i. π_RF (π_RF1) is a radio-frequency π pulse driving the nuclear spin transition between states $|m_S = 0, m_I = 1\rangle$ and $|m_S = 0, m_I = 0\rangle$ (transition between states $|m_S = 0, m_I = 0\rangle$ and $|m_S = 0, m_I = -1\rangle$). The measured photoluminescence intensity after each sequence is denoted by S_1, S_2, R_1, or R_2.

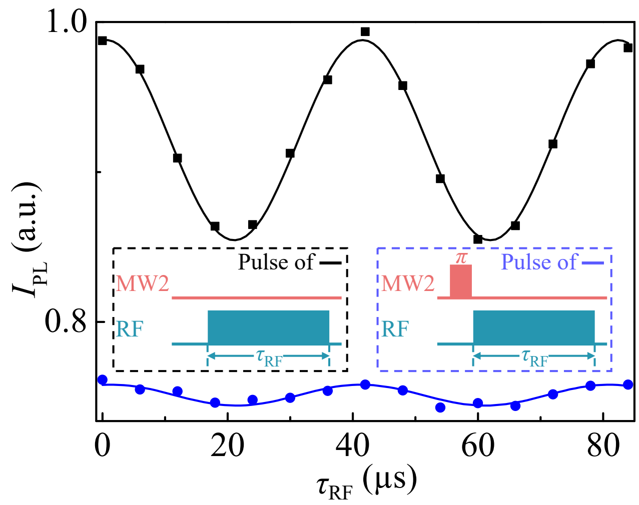

Supplementary Figure 9: **Experimental measurement of the polarization of the NV electron spin.** The polarization α is extracted from the ratio of the two nutation amplitudes to be 0.83(2).

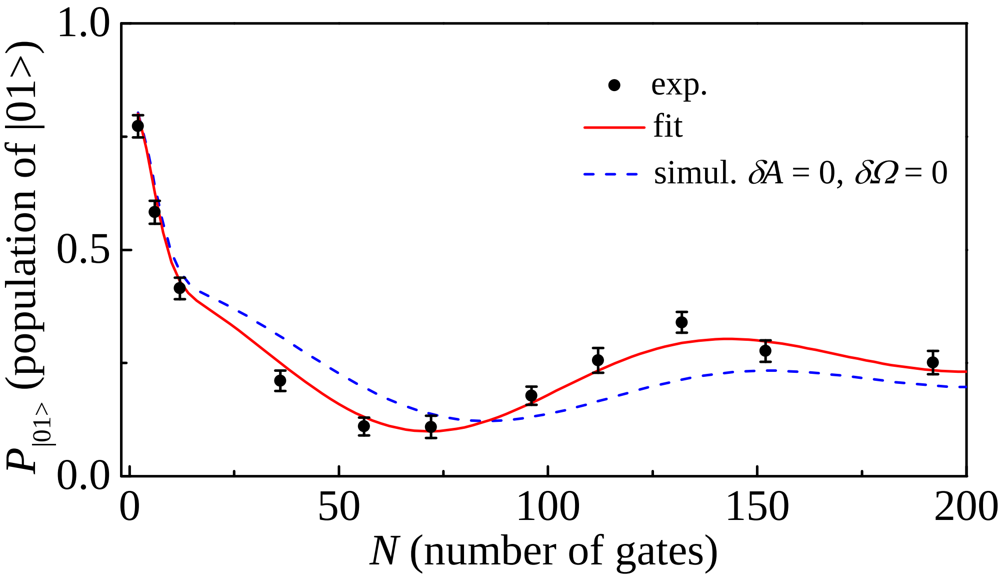

Supplementary Figure 10: **Extraction of the practical Hamiltonian.** The experimentally measured $P_{|01\rangle}$ is shown as the black circles. The practical Hamiltonian is extracted by fitting the experimental data. The fitted $P_{|01\rangle}$ is shown as the red solid line. Best-fit values of δA and $\delta\Omega$ are $\delta A = 0.008(1)$ MHz and $\delta\Omega = 0.068(1)$ MHz. For comparison, the blue dashed line shows the simulated result with $\delta A = 0$ and $\delta\Omega = 0$.

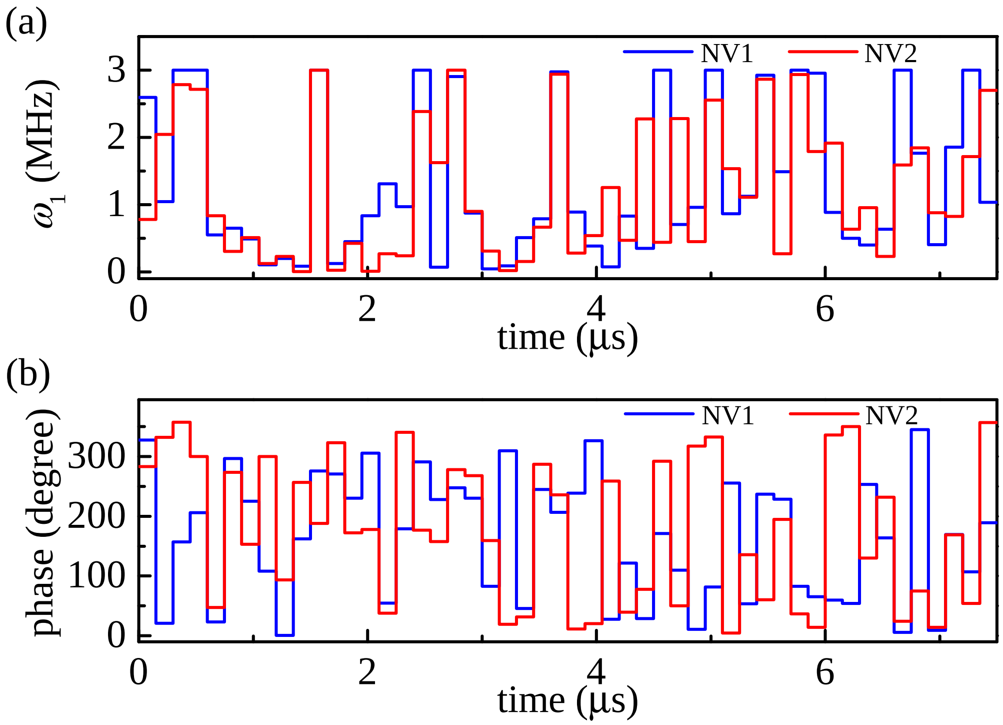

Supplementary Figure 11: **Pulse sequence for the CNOT gate in NV-NV coupled system.** (a) Amplitudes of the control microwave fields. (b) Phases of the control microwave fields.

Supplementary Table 1: **Summarization of the results of single-qubit randomized benchmarking.** Average gate fidelity (F_{a}) and error per gate (ε_{g}) is shown for naive, five-piece SUPCODE, BB1, and BB1inC pulses.

Pulse sequence	F_{a}	ε_{g}
naive	0.99968(6)	$3.2(6)\times10^{-4}$
five-piece SUPCODE	0.99916(8)	$8.4(8)\times10^{-4}$
BB1	0.999945(6)	$5.5(6)\times10^{-5}$
BB1inC	0.999952(6)	$4.8(6)\times10^{-5}$

Supplementary Note 1: Hamiltonian of the NV system

The NV center includes a substitutional nitrogen atom and a vacancy in the nearest-neighbor lattice position. In our experiment, a static magnetic field, $B_0 = 513$ G, is applied along the NV symmetry axis ([1 1 1] crystal axis). The Hamiltonian of the NV center can be written as

$$H_{\text{NV}} = 2\pi(DS_z^2 + \omega_S S_z + PI_z^2 - \omega_I I_z) + H_{\text{hf}}, \tag{1}$$

where $\omega_S = -\gamma_{\text{e}} B_0/2\pi$ ($\omega_I = \gamma_{\text{N}} B_0/2\pi$) is the Zeeman splitting of the electron (^{14}N nuclear) spin, γ_{e} (γ_{N}) is the electronic (^{14}N nuclear) gyromagnetic ratio, S_z and I_z are the electron and nitrogen nuclear spin operators, respectively. The zero field splitting $D = 2870$ MHz and the nuclear quadrupolar splitting $P = -4.95$ MHz. The hyperfine interaction between the NV electron spin and the ^{14}N nuclear spin is

$$H_{\text{hf}} = 2\pi[A_\perp(S_x I_x + S_y I_y) + AS_z I_z], \tag{2}$$

The strength of the hyperfine interaction is about 2 MHz. Because of the strong zero field splitting and Zeeman splitting terms of the electron spin, the effect of the interaction term $S_x I_x + S_y I_y$ is strongly suppressed and can be neglected. $A = -2.16$ MHz is determined via CW ESR experiment. In the secular approximation, the Hamiltonian is

$$H_{\text{NV}} = 2\pi(DS_z^2 + \omega_S S_z + AS_z I_z + PI_z^2 - \omega_I I_z), \tag{3}$$

The electron (nuclear) spin states $|m_S = 0\rangle$ and $|m_S = -1\rangle$ ($|m_I = 0\rangle$ and $|m_I = +1\rangle$) are encoded as the electron (nuclear) spin qubit.

Microwave (MW) and radio-frequency (RF) pulses are used to manipulate the two-qubit system. The frequency of MW and RF pulses are f_{MW} and f_{RF} , respectively. When MW pulses are applied, the total Hamiltonian becomes

$$H = H_{\text{NV}} + H_{\text{C}}, \tag{4}$$

with

$$H_{\text{C}} = 2\pi\sqrt{2}\omega_1 \cos(2\pi f_{\text{MW}} t + \phi) S_x, \tag{5}$$

where ϕ is the phase of the MW pulse, ω_1 is the amplitude of the MW pulse.

The Hamiltonian can be transformed into the rotating frame as

$$H_{\text{rot}} = U_{\text{trans}} H U_{\text{trans}}^\dagger - iU_{\text{trans}} \frac{dU_{\text{trans}}^\dagger}{dt}, \tag{6}$$

with

$$U_{\text{trans}} = e^{i2\pi f_{\text{MW}} t S_z^2} e^{-i2\pi f_{\text{RF}} t I_z^2}. \tag{7}$$

With rotating-wave approximation, the Hamiltonian in the rotating frame can be simplified as

$$H_{\text{rot}} = 2\pi(\omega_S(S_z + S_z^2) - \omega_I(I_z - I_z^2) + A(S_z^2 + S_z I_z)) + H_{\text{C, rot}}, \tag{8}$$

where

$$H_{\text{C, rot}} = 2\pi[\delta\Omega S_z^2 + \omega_1/\sqrt{2}(\cos\phi H_x + \sin\phi H_y)], \tag{9}$$

with

$$H_x = \frac{1}{\sqrt{2}} \begin{pmatrix} 0 & 1 & 0 \\ 1 & 0 & 1 \\ 0 & 1 & 0 \end{pmatrix} \otimes \mathbb{I}, \tag{10}$$

$$H_y = \frac{1}{\sqrt{2}} \begin{pmatrix} 0 & -i & 0 \\ i & 0 & i \\ 0 & -i & 0 \end{pmatrix} \otimes \mathbb{I}, \tag{11}$$

, $\delta\Omega = D - \omega_S - A - f_{\text{MW}}$, $f_{\text{RF}} = -P + \omega_I$, and $\mathbb{I}$ representing 3×3 identity matrix.

Supplementary Note 2: Calculation of average gate fidelity

The average gate fidelity between a quantum operation ξ and a target unitary quantum gate U is defined as

$$F_{\text{a}}(\xi, U) = \int d\psi \langle\psi|U^\dagger \xi(|\psi\rangle\langle\psi|)U|\psi\rangle, \tag{12}$$

where the integral is over the uniform measure $d\psi$ on state space, normalized so $\int d\psi = 1$[1].

In the single-qubit case, the average gate fidelity can be derived to be[2]

$$F_{\text{a}}^{(1)}(\xi, U) = \frac{1}{2} + \frac{1}{12} \sum_{j=x,y,z} tr(U\sigma_j U^\dagger \xi(\sigma_j)), \tag{13}$$

where σ_x, σ_y, and σ_z are Pauli matrices.

Quantum optimal control method [3] is used to design the pulse sequence of CNOT gate. To calculate the average gate fidelity of this CNOT gate, Eqn. 12 is generalized so that the integral is on the two-qubit space[4]. The nuclear spin is much less sensitive to the external magnetic noise than the electron spin and the GRAPE pulse sequence contains only microwave pulses, so the decoherence during the operation mainly comes from the static distributions of δ_0 and δ_1 for the electron spin qubit. Then the operation can be expressed as

$$\xi(|\psi\rangle\langle\psi|) = \int d\delta_0 \int d\delta_1 P_0(\delta_0)P_1(\delta_1)U_{\text{seq}}(\delta_0,\delta_1)|\psi\rangle\langle\psi|U^\dagger_{\text{seq}}(\delta_0,\delta_1), \tag{14}$$

where $U_{\text{seq}}(\delta_0,\delta_1)$ is the calculated two-qubit evolution according to the pulse sequence, with the errors δ_0 and δ_1 considered in Hamiltonian. Substituting Eqn. 14 into Eqn. 12 yields the average gate fidelity between the operation ξ and the target CNOT gate U_{CNOT},

$$F_{\text{a}}(\xi, U_{\text{CNOT}}) = \frac{1}{d(d+1)} \int d\delta_0 \int d\delta_1 P_0(\delta_0)P_1(\delta_1)(tr(MM^\dagger) + |tr(M)|^2), \tag{15}$$

with $d = 4$ and

$$M = U^\dagger_{\text{CNOT}} U_{\text{seq}}(\delta_0,\delta_1). \tag{16}$$

It can be easily obtained from Eqn. 15 that the fidelity of operation ξ without the effect of the noise (δ_0 and δ_1) can be written as

$$F_{\text{seq}} = \frac{1}{d(d+1)}[tr(MM^\dagger) + |tr(M)|^2], \tag{17}$$

where the values of δ_0 and δ_1 are zero.

Supplementary Note 3: High fidelity single-qubit quantum gates

Considering a single-qubit gate corresponding to a rotation of angle θ around the x axis on the Bloch sphere, such a gate can be realized by evolution under the effective Hamiltonian $H_{\text{ideal}} = 2\pi\omega_1 \mathbf{n}\cdot\mathbf{S}$, where $\mathbf{S} = (S_x, S_y, S_z)$ is the spin vector operator of the qubit, $\mathbf{n}$ is a three-dimensional vector, and the strength ω_1 is a real parameter. The average gate fidelity is limited by interaction of the qubit with environment and fluctuation of the control field. We consider the model

where the Hamiltonian for rotation about the x axis under practical conditions is described as $H_{\text{prac}} = 2\pi\delta_0 S_z + 2\pi(\omega_1 + \delta_1)[\cos\delta\phi S_x + \sin\delta\phi S_y]$. The error δ_0 in the Hamiltonian is due to the interaction of the qubit with environment, the error δ_1 is due to fluctuation of the control field strength and phase error $\delta\phi$ is caused by the imperfect microwave pulse generation. Phase error can be efficiently eliminated by pulse fixing technique (detailed in Section) and we take it as of zero value in this section. We consider the case where both δ_0 and δ_1 vary in a timescale much longer than that of quantum gates. In this case δ_0 and δ_1 are taken as quasi-static random constants.

Supplementary Figure 1a shows the performance of the gate by simply applying a naive rectangular pulse. Here the gate $(\pi/2)_0$ (we denote the rotation of an angle θ around the axis in the equatorial plane with azimuth ϕ as $(\theta)_\phi$) is taken as an example. The average gate fidelity of $(\pi/2)_0$ is calculated with respect to different values of δ_0 and δ_1. The naive pulse is vary sensitive to the errors δ_0 and δ_1, with leading orders of both errors preserved in the evolution operator (corresponding to second orders in the average gate fidelity). This corresponds with the small region of high average gate fidelity shown in the lower panel of Supplementary Figure 1a.

Supplementary Figure 1b shows a type of dynamically corrected gate, five-piece SUPCODE[5]. The pulse sequence is depicted as $\tau_1 - (\theta/2)_0 - \tau_3 - (\theta/2)_0 - \tau_1$ for $\theta \in (2\pi, 3\pi)$. Here $\tau_1 = \csc\theta(1 - 2\cos\frac{\theta}{2} + \cos\theta + \sqrt{4 - 8\cos\frac{\theta}{2} + 4\cos\theta + \theta\sin\theta})$ and $\tau_3 = -2(\tau_1\cos\frac{\theta}{2} + \sin\frac{\theta}{2})$ are durations when control field is off. Under the five-piece SUPCODE pulse, up to second order of δ_0 can be canceled (corresponding to sixth order preserved in the average gate fidelity). In the lower panel of Supplementary Figure 1b, the average gate fidelity of $(2.5\pi)_0$ (equivalent to $(\pi/2)_0$ in the single-qubit case) is shown as an example. The region of high average gate fidelity is largely extended in the axis of δ_0, compared with that by the naive pulae.

In Supplementary Figure 1c a type of composite pulse, BB1[6], is shown. The pulse sequence is $(\theta/2)_0 - (\pi)_\phi - (2\pi)_{3\phi} - (\pi)_\phi - (\theta/2)_0$, with $\phi = \arccos(-\theta/4\pi)$. Under the BB1 pulse, up to second order of δ_1 is canceled in the evolution operator (corresponding to sixth order preserved in

the average gate fidelity). The average gate fidelity of $(\pi/2)_0$ with the BB1 pulse is shown in the lower panel of Supplementary Figure 1c. It exhibits a larger region of high fidelity in the axis of δ_1. Thus suppressing the δ_1 error by applying the BB1 pulse, in combination with a proper selection of control field strength ω_1 to suppress the δ_0 error, can contribute to realization of a high fidelity (e.g. 0.9999).

Supplementary Figure 1d shows a pulse sequence suppressing both the δ_0 and δ_1 errors simultaneously. The sequence is designed by incorporating the BB1 pulse within the CORPSE pulse, and is named BB1inC here for short. There are similar pulse sequences to suppress the δ_0 and δ_1 error simultaneously[7]. The BB1inC sequence is depicted as $(\theta/2)_0 - (\pi)_\phi - (2\pi)_{3\phi} - (\pi)_\phi - (\theta_3)_0 - (\theta_2)_\pi - (\theta_1)_0$, where $\phi = \arccos(-\theta/4\pi)$, $\theta_1 = \theta/2 - \arcsin(\sin(\theta/2)/2)$, $\theta_2 = 2\pi - 2\arcsin(\sin(\theta/2)/2)$, and $\theta_3 = 2\pi - \arcsin(\sin(\theta/2)/2)$. Leading orders of both the δ_0 and δ_1 errors are canceled in the evolution operator. The lower panel of Supplementary Figure 1d shows the average gate fidelity of $(\pi/2)_0$ with the BB1inC pulse. The region of high fidelity is much larger, extended in both axes of δ_0 and δ_1.

Recently the robustness of composite pulse sequences against time-dependent noise is analyzed[8]. It is shown that composite pulses may also be successfully employed in the presence of time-dependent noise. The robustness against static as well as time-dependent noise enables composite pulse an effective method to improve single-qubit gate fidelity.

Supplementary Note 4: Quantum optimal control method for designing the CNOT gate

GRAPE is a type of quantum optimal control method. It can be utilized to design control sequence to realize a target gate with high fidelity. The control sequence contains N piece of pulses, with the amplitude and phase of each piece being different. The total Hamiltonian of the kth pulse in the rotating frame is (see Eqn. 8 and Eqn. 9)

$$H_{\text{rot},k} = 2\pi(\omega_S(S_z + S_z^2) - \omega_I(I_z - I_z^2) + A(S_z^2 + S_z I_z)) + H_{\text{C, rot},k}, \quad (18)$$

with

$$H_{\text{C, rot},k} = 2\pi\omega_{1,k}/\sqrt{2}(\cos\phi_k H_x + \sin\phi_k H_y), \quad (19)$$

where ϕ_k is the phase of the kth pulse, $\omega_{1,k}$ is the amplitude of the kth microwave pulse and $\delta\Omega = 0$.

The evolution operator under the kth pulse is written as

$$U_k = e^{-iH_{\text{rot},k} t_k}, \tag{20}$$

where t_k is the duration of the kth pulse. The total evolution under the entire sequence is

$$U_{\text{tot}} = \prod_{k=N}^{1} U_k. \tag{21}$$

The time duration of each pulse, t_k, is set to be equal value τ. The two-qubit evolution operator can be described as

$$U_{\text{seq}}(\{\omega_{1,k}, \phi_k\}) = \mathcal{P} \prod_{k=N}^{1} e^{-iH_{\text{rot},k}(\{\omega_{1,k},\phi_k\})\tau} \mathcal{P}, \tag{22}$$

where $\mathcal{P}$ is the projection operator on the two-qubit subspace.

The target gate is the CNOT gate,

$$U_{\text{CNOT}} = \begin{pmatrix} 0 & 0 & 1 & 0 \\ 0 & 1 & 0 & 0 \\ 1 & 0 & 0 & 0 \\ 0 & 0 & 0 & 1 \end{pmatrix}. \tag{23}$$

The performance function of the GRAPE algorithm is the fidelity F_{seq}, which is a function of $\{\omega_{1,k}, \phi_k\}$. The values of $\{\omega_{1,k}, \phi_k\}$ are initialized with random numbers within the experimental conditions. The performance function are maximized by iteration. Within each iteration, the value of $F_{\text{seq}}(\{\omega_{1,k}, \phi_k\})$ as well as its derivative to $\omega_{1,k}$ and ϕ_k is calculated, then the value of $\omega_{1,k}$ (ϕ_k) is replaced by the result of its previous value plus the derivative multiplied by a proper coefficient.

This method works well for designing a sequence with high fidelity, if errors due to qubit-environment interaction and control field fluctuation are not taken into account. However, our aim is to realize quantum gates, which are not only of high fidelity, but also being robust to the errors. A method has been presented[3] to design pulse sequence which is robust against the inhomogeneity

of ω_1. Herein, we generalize this method to design pulse sequence, which is not only robust against the inhomogeneity δ_1, but also is insensitive to the dephasing noise δ_0. The performance function of the modified GRAPE is defined as $F_{\mathrm{a}}(\varepsilon, U_{\mathrm{CNOT}})$ (see Eqn. 15). The new performance function is maximized by iteration of the GRAPE algorithm. In practical implementation of the modified algorithm, the integral in Eqn. 15 is replaced by sum of discrete points. We find that three points of δ_0 (δ_1) are enough.

Supplementary Figure 2a shows the amplitude and phase of the designed pulse sequence. The sequence consists of twelve pieces of pulse. The duration of each pulse is 58 ns. Without considering the errors δ_0 and δ_1, the sequence produces a two-qubit operation U_{cal},

$$U_{\mathrm{cal}} = e^{1.5974i} \begin{pmatrix} -0.0060+0.0032i & 0 & 1-0.0013i & 0 \\ 0 & 0.9996 & 0 & 0.0154-0.0249i \\ 0.9999+0.0119i & 0 & 0.0059+0.0032i & 0 \\ 0 & -0.0146-0.0253i & 0 & 0.9991+0.0316i \end{pmatrix}, \tag{24}$$

The fidelity of U_{cal} is 0.9995. Supplementary Figure 2b shows the robustness of the sequence against the errors δ_0 and δ_1. When the experimental distributions $P_0(\delta_0)$ and $P_1(\delta_1)$ (which are determined from the experiments) are considered, the sequence provides an average gate fidelity of 0.9927.

Supplementary Note 5: Alignment of the magnetic field, Creation of a solid immersion lens, Ultra-broadband coplanar waveguide

We used the fluorescence dependence on the misalignment angle to align the magnetic field. According to the literature[9], the fluorescence of NV center is sensitive to misalignment angle of the NV axis from a magnetic field $\mathbf{B}_0$ when the magnitude is approximately 513 G. The difference in fluorescence counts is still noticeable even when the misalignment angle is only 0.5°. In our experiment, the fluorescence count was the same (within counting errors) for $B_0 \approx 0\,G$ and $B_0 \approx 513$ G. So we estimate the misalignment angle to be within $0.5°$.

All measurements in our experiment are based on detection of the NV photoluminescence. Much of the photoluminescence is lost at the diamond surface due to internal reflection. The problem can be overcome by creating a solid immersion lens (SIL)[10]. We created a SIL in the diamond around an NV center (Supplementary Figure 3). The SIL increases the PL rate to about 400 kcounts s^{-1}.

In the experiment, manipulation of qubits is achieved by microwave (MW) and radio-frequency (RF) pulses, which are applied through a coplanar waveguide (CPW). The ultra-broadband CPW is designed and fabricated. Supplementary Figure 4 shows scattering parameters of the CPW. Up to 15 GHz, the S21 parameter is larger than -3 dB, and the S11 parameter is about or less than -10 dB. Such wide bandwith ensures that there is almost no extra distortion of microwave / RF pulses with this CPW.

Supplementary Note 6: Correction of microwave pulse distortions

The imperfect devices generate microwave pulses with non-ideal amplitudes and phases. The imperfection of microwave pulses sent to the NV centers are carefully corrected with pulse fixing technique[11]. Supplementary Figure 5a shows the main instruments for the microwave pulse generation. The pulses are generated by an arbitrary waveform generator (M8190A, Keysight), and amplified with a power amplifier (ZHL-30W-252-S+, Mini Circuits). The imperfections in the instruments cause distortions of the microwave pulses, which may dramatically decrease fidelity of quantum gates. An oscilloscope (DSO-X 92004Q) is used to sample the microwave pulses. The pulse distortions are then corrected by predistorting the pulses in the right way that the predistortions cancel with the distortions by the imperfections of instruments.

Microwave phase correction. Supplementary Figure 5b shows the distortion of the microwave pulse phase with / without the correction. It is clear that there is no significant distortion of the microwave pulse phase with the correction, as shown in the right panel of Supplementary Figure 5b. In Supplementary Figure 5c, we compare the average gate fidelity theoretically with the microwave pulse with and without the phase correction. Because of the pulse distortions, the

BB1inC $\pi/2$ gate becomes less error-resilient, without any region of fidelity higher than 0.9999 (see the left panel of Supplementary Figure 5c). The result in the right panel of Supplementary Figure 5c shows a region with fidelity higher than 0.9999 with the phase correction.

Microwave amplitude correction. In the two-qubit experiment, microwave and radio-frequency pulses are combined with a diplexer (Marki DPX-1). We find that the leakage and reflection of the diplexer ports cause extra distortions of the microwave pulses. Supplementary Figure 6a shows that there are distortion of the microwave amplitude. After inserting a 10 dB attenuator between the microwave components to suppress the leakage and reflection, the waveform of the pulse sequence is improved to be close to the ideal case, as shown in Supplementary Figure 6b.

The distortions of amplitude shown in Supplementary Figure 6b can be further corrected by pulse fixing technique. Similar to that shown in Supplementary Figure 5a, the distortions are recorded by an oscilloscope, and then fed back to the arbitrary waveform generator so that the distortions are minimized. Supplementary Figure 7 shows the comparison of the pulse waveforms without and with amplitude correction.

Supplementary Note 7: Normalization of the experimental data

In the single-qubit experiment, the normalization is carried out by performing a nutation experiment[12]. The normalized data corresponds to the population of $|0\rangle$ for the final state.

In the two-qubit experiment, the population of $|m_S = 0, m_I = 1\rangle$ ($P_{|01\rangle}$) for the final state is obtained by normalization. According to the Ref. 13, each occupied energy level contributes to the measured photoluminescence intensity (I_{PL}) with a different PL rate and these different PL rates are measured and used to determine the population of the levels with several sequences. Herein we introduce an alternative method for normalization. The pulse sequences for the normalization

are shown in Supplementary Figure 8. The measured I_{PL} is

$$I_{\text{PL}} = \sum_{|k\rangle} \beta_{|k\rangle} P_{\rho,|k\rangle}, \tag{25}$$

where $|k\rangle$ denotes the nine energy levels of the NV center ($|m_S, m_I\rangle$ with $m_S = 0, \pm 1$ and $m_I = 0, \pm 1$), $P_{\rho,|k\rangle}$ is the population of $|k\rangle$ for the state ρ, β_k is the photoluminescence intensity if the state is $|k\rangle$.

In Supplementary Figure 8, ρ_{i} denotes the initialized state after initializing laser pulse, ρ_{f} denotes the final state after applying control sequence to ρ_{i}. The RF (RF1) π pulse exchanges the population of $|m_S = 0, m_I = 1\rangle$ and $|m_S = 0, m_I = 0\rangle$ ($|m_S = 0, m_I = 0\rangle$ and $|m_S = 0, m_I = -1\rangle$). The measured I_{PL} after the four sequences (S_1, S_2, R_1, and R_2, respectively) satisfy

$$S_1 - S_2 = (\beta_{|0,1\rangle} - \beta_{|0,-1\rangle})(P_{|0,1\rangle} - P_{|0,-1\rangle}), \tag{26}$$

$$R_1 - R_2 = (\beta_{|0,1\rangle} - \beta_{|0,-1\rangle})(P_{\rho_{\text{i}},|0,1\rangle} - P_{\rho_{\text{i}},|0,-1\rangle}), \tag{27}$$

where $P_{|k\rangle}$ ($P_{\rho_{\text{i}},|k\rangle}$,) is the population of $|k\rangle$ for ρ_{f} (ρ_{i}). After the initializing laser pulse, the electron spin is polarized with coefficient α, and the nuclear spin is almost completely polarized. Thus we have $P_{\rho_{\text{i}},|0,1\rangle} = \alpha$ and $P_{\rho_{\text{i}},|0,-1\rangle} = 0$. The state $|m_S = 0, m_I = -1\rangle$ remains idle during the control sequence, thus $P_{|0,-1\rangle} = 0$. The population of $|m_S = 0, m_I = 1\rangle$ for the final state can be derived as

$$P_{|01\rangle} = \alpha \frac{S_1 - S_2}{R_1 - R_2}, \tag{28}$$

Supplementary Note 8: Measuring the polarization of the NV electron spin

The measurement of the polarization described here is similar to that described before[13]. Supplementary Figure 9 shows the results and pulse sequences used in the measurement. We first recorded the nuclear Rabi oscillation by driving the $|m_S = 0, m_I = 1\rangle$ and $|m_S = 0, m_I = 0\rangle$ transition. The nuclear spin is almost completely polarized. The amplitude of this nuclear Rabi oscillation is proportional to the polarization α of the electron spin, with

$$A1 = (\beta_{|0,1\rangle} - \beta_{|0,0\rangle})\alpha. \tag{29}$$

Secondly, another nuclear Rabi oscillation is recorded after a MW2 π pulse. The MW2 π pulse exchanges the population of $|m_S = 0, m_I = 1\rangle$ and $|m_S = -1, m_I = 1\rangle$. The amplitude of this nuclear Rabi oscillation is proportional to the population of $|m_S = -1, m_I = 1\rangle$ for the initialized state.

$$A2 = (\beta_{|0,1\rangle} - \beta_{|0,0\rangle})(1 - \alpha)/2, \tag{30}$$

Then the polarization α can be obtained with

$$\alpha = \frac{1}{1 + 2\frac{A_2}{A_1}}, \tag{31}$$

With the results shown in Supplementary Figure 9, we estimated the polarization of the NV electron spin to be $\alpha = 0.83(2)$.

Supplementary Note 9: Measurement of the average gate fidelity

We first describe the method for measuring the average gate fidelity of single-qubit gates. The average gate fidelity of single-qubit gates are measured with randomized benchmarking (RB) method[14]. Unlike that with quantum process tomography, the measured fidelity with RB method is not limited by errors in state preparation and measurement. The qubit is initialized to $|0\rangle$, then a predetermined sequence of randomized computational gates is applied. Each computational gate consists of a Pauli gate followed by a (non-Pauli) Clifford gate. Pauli gates are randomly chosen to rotate the qubit about the $\pm x$, $\pm y$, or $\pm z$ axes for an angle π on the Bloch sphere, or to be a $\pm I$ identity gate; Clifford gates are randomly chosen to rotate about the $\pm x$ or $\pm y$ axes for an angle $\pi/2$. The gate sequence is followed by a final Clifford gate chosen to ensure that the final qubit state is $|0\rangle$ if all the gates are ideal. The fidelity of the final state ρ_{f}, $F = \langle 0|\rho_{\mathrm{f}}|0\rangle$, is measured. The measured final state fidelity is averaged over different random sequences. The averaged fidelity, $\overline{F}$, is fitted with Eqn. 32

$$\overline{F} = 1/2 + 1/2(1 - d_{\mathrm{if}})(1 - 2\varepsilon_{\mathrm{g}})^l, \tag{32}$$

where l is the number of computational gates, ε_{g} is the average error per gate, and d_{if} describes errors in state preparation and measurement. The average gate fidelity is

$$F_{\mathrm{a}} = 1 - \varepsilon_{\mathrm{g}}, \tag{33}$$

In the experiment, $\pm x, \pm y$ rotations are realized by proper microwave settings, and $\pm z$ rotations are implemented by a rotation of the logical frame of the qubit for the subsequent pulses[15–17].

For the naive pulse, each Clifford gate is performed by a rectangular $\pi/2$ pulse and each Pauli gate by a rectangular π pulse; For the five-piece SUPCODE pulse, each Clifford gate is performed by a five-piece SUPCODE 2.5π pulse (equivalent to $\pi/2$ in the single-qubit case, see Section) and each Pauli gate by a pair of five-piece SUPCODE 2.5π pulses; For the BB1 (BB1inC) pulse, each Clifford gate is performed by a BB1 (BB1inC) $\pi/2$ pulse and each Pauli gate by a BB1 (BB1inC) π pulse (see Section).

The RB results for naive, five-piece SUPCODE, BB1 and BB1inC pulses are shown in Fig. 2(b) in the main text and summarized in Supplementary Table **1**. The measured average gate fidelities are 0.99968(6), 0.99916(8), 0.999945(6) and 0.999952(6), respectively.

In the following we describe the method for measuring the average gate fidelity of two-qubit CNOT gate. The average gate fidelity of CNOT can be measured with Eqn. 15. This requires the full knowledge of the quantum operation ξ, which is usually very difficult to be obtained. Quantum process tomography has been developed to characterize the quantum gates. However, this procedure requires a number of measurements that scale exponentially with the number of qubits, and the measured process matrix is sensitive to errors in state preparation and measurement. Randomized bechmarking and related techniques are developed to obtain the average gate fidelities. However, in the hybrid system composed of electron and nuclear spins, single-qubit gates on the nuclear spin cost longer time than the electron coherence time. The error of gates on the nuclear spin will dominate the fidelity decay in randomized benchmarking, and the gate fidelity of CNOT can not be precisely determined this way.

Herein, we present a method to estimate the average fidelity of CNOT gate. We determine the fidelity by repeated application of the CNOT gates on the system. A wealth of information can be obtained by studying the state dynamics under repeated application of quantum gates[18]. In Ref.

19, CNOT gates were repeatedly applied on the input state generated by $X_{-\pi/2} \otimes I$, and the fidelity F_s of final states were measured. The fidelity F_s decays as the number of the CNOT gate, N, is increased. The maximum value of N was 12 in that work. By assuming that the decay obeys an exponential model, the gate fidelity F_g can be extracted.

The pulse sequence used in our experiment is shown in the inset of Fig. 4e in the main text. The initial state of the two-qubit system is prepared by applying a RF $\pi/2$ pulse after the initial laser pulse. Then N, which is even, times of repeated CNOT gates are applied. Finally, the population of state $|01\rangle$ ($P_{|01\rangle}$) after another RF $\pi/2$ is measured. Up to 192 CNOT gates are applied, the dynamics of $P_{|01\rangle}$, however, does not obey a simple exponential decay. As shown in Supplementary Figure **10**, the measured $P_{|01\rangle}$ oscillates while decaying with N. In our experiment, the nuclear spin qubit is extremely 'clean' due to being insensitive to the external noises. The CNOT gate designed by quantum optimal control method consists of microwave pulses only. Thus the decay is due to the static fluctuation of δ_0 and δ_1, while the oscillation is mainly due to the deviation of the experimental operation from the ideal CNOT gate.

We simulated the dynamics of $P_{|01\rangle}$ based on the Hamiltonian H_rot, the pulse sequence (shown in Supplementary Figure **2**) and the distributions of δ_0 and δ_1. The simulated dynamics of $P_{|01\rangle}$ is presented as the blue dashed line in Supplementary Figure **10**. The deviation between the experimental result and the simulated result shown in Supplementary Figure **10**, is mainly due to the difference between the H_rot and the practical one $H_\text{rot,prac}$ in the experiment. The microwave frequency does not equal the resonance frequency exactly, so the off-resonance term $\delta\Omega$ is of a prior unknown nonzero value. The value of hyperfine coupling strength $A = -2.16$ MHz, which is used for pulse sequence designing, can also deviate slightly from the practical one A_exp. We denote this difference as $\delta A = A_\text{exp} - A$. The practical Hamiltonian $H_\text{rot,prac}$ can be extracted by fitting the experimental data. The fitting procedure is accomplished with Matlab. The best-fit values of the parameters are $\delta A = 0.008(1)$ MHz and $\delta\Omega = 0.068(4)$ MHz, with the errors being the uncertainty within 95% confidence. The extracted values of δA and $\delta\Omega$ are much smaller than the value of CW spectrum's linewidth. The fitting result, which agrees with the experimental data

well, is shown as the red solid line in Supplementary Figure **10**. With the values of δA and $\delta\Omega$, we can determine the fidelity of CNOT gate according to Eqn. 15. The derived average gate fidelity is 0.9920(1), where the error is due to the uncertainty of δA and $\delta\Omega$.

Supplementary Note 10: Robust and precise optimal control method on NV-NV system

We have demonstrated a high fidelity CNOT gate at fault-tolerant threshold, taking the NV electron spin and ^{14}N nuclear spin as qubits. The CNOT gate is designed with modified optimal control method. This method can also be used to design robust and precise quantum gates on NV-NV coupled system, a key ingredient for scalable quantum computation using diamond.

The static Hamiltonian of two coupled NV centers can be described as

$$H_0 = H_{\text{NV,1}} + H_{\text{NV,2}} + H_{\text{int}}, \tag{34}$$

with

$$H_{\text{NV,1}} = 2\pi D S_{z1}^2 - \gamma_e \boldsymbol{B}_{0,1} \cdot \boldsymbol{S}_1, \tag{35}$$

$$H_{\text{NV,2}} = 2\pi D S_{z2}^2 - \gamma_e \boldsymbol{B}_{0,2} \cdot \boldsymbol{S}_2, \tag{36}$$

$$H_{\text{int}} = 2\pi \boldsymbol{S}_1 \cdot \mathbb{C} \cdot \boldsymbol{S}_2, \tag{37}$$

where $\boldsymbol{S}_1$ and $\boldsymbol{S}_2$ are the spin operators of individual NV centers, NV 1 and NV 2, respectively. The zero filed splitting is D =2870 MHz. The coupling tensor between NV 1 and NV 2 is denoted as $\mathbb{C}$. The static magnetic field applied on NV 1 (NV 2) is $\boldsymbol{B}_{0,1}$ ($\boldsymbol{B}_{0,2}$).

The system can be controlled by oscillating magnetic fields. The corresponding control Hamiltonian is

$$H_{\text{C, NV-NV}}(t) = -\gamma_{\text{e}} \sum_m \cos[2\pi f_m t + \phi_m(t)] \boldsymbol{B}_{1,m}(t) \cdot (\boldsymbol{S}_1 + \boldsymbol{S}_2), \tag{38}$$

where f_m are the carrier frequencies of the control fields, $\boldsymbol{B}_{1,m}$ contain the amplitudes $B_{1,m} = |\boldsymbol{B}_{1,m}|$ and the polarization $\boldsymbol{u}_m = \boldsymbol{B}_{1,m}/B_{1,m}$. The amplitudes $B_{1,m}$ and the phases ϕ_m can be changed in time to steer the system.

We turn to a rotating frame, in which the Hamiltonian is

$$H'(t) = e^{i(H_{\text{NV},1}+H_{\text{NV},2})t}[H_{\text{int}} + H_{\text{C, NV-NV}}(t)]e^{-i(H_{\text{NV},1}+H_{\text{NV},2})t}, \tag{39}$$

The evolution operator with a time duration T is

$$U_{\text{e}}(T) = \mathcal{T}e^{-i\int_0^T dtH'(t)}, \tag{40}$$

where $\mathcal{T}$ is the time-ordering operator.

Similar to that described in Section , the pulse sequence for a target two-qubit unitary gate U can be designed by maximizing the performance function

$$F'_{\text{seq}} = \frac{1}{d(d+1)}[tr(M'M'^{\dagger}) + |tr(M')|^2], \tag{41}$$

with d =4 and

$$M' = U^{\dagger}\mathcal{P}U_{\text{e}}(T)\mathcal{P}, \tag{42}$$

where $\mathcal{P}$ is projection operator on the two-qubit subspace.

Considering the quasi-static noises from environment and the control fields, the control Hamiltonian in Eqn. 38 is replaced by

$$H_{\text{C, err, NV-NV}}(t) = 2\pi\delta_{0,1}S_{z1}+2\pi\delta_{0,2}S_{z2}-\gamma_{\text{e}}\sum_m \cos[2\pi f_m t+\phi_m(t)](1+\delta_{1,m,\text{ rel}})\boldsymbol{B}_{1,m}(t)\cdot(\boldsymbol{S}_1+\boldsymbol{S}_2), \tag{43}$$

With $H_{\text{C, NV-NV}}(t)$ replaced by $H_{\text{C, err, NV-NV}}(t)$, it is straight forward to calculate the evolution operator $U_{\text{e, err}}(T)$ and gate fidelity $F'_{\text{seq, err}}$ with quasi-static noises described by $\delta_{0,1}$, $\delta_{0,2}$, and $\delta_{1,m,\text{ rel}}$. Then the performance function is defined by integrating $F'_{\text{seq, err}}$ over distributions of $\delta_{0,1}$, $\delta_{0,2}$, and $\delta_{1,m,\text{ rel}}$. By maximizing the performance function, pulse sequence for target U can be designed to be robust against the noises.

We take the optimization of pulse sequence for a robust and precise CNOT gate as an example. The static magnetic field applied on each NV center is aligned along the NV symmetry axis,

and the NV centers can be individually addressable by application of gradient magnetic field. The coupling strength is taken to be 100 kHz, corresponding to a distance of about 8 nm between two NV centers[20]. According to Ref. 21, a magnetic-field gradient of 12 G nm^{-1} is available, corresponding to a difference of more than 200 MHz between the NV centers' resonant frequencies. The spin states $|m_S = 0\rangle$ and $|m_S = -1\rangle$ of NV 1 (NV 2) are encoded as $|0\rangle$ and $|1\rangle$ of qubit 1 (qubit 2). Microwave pulses with two frequencies, which are resonant frequencies for the two qubits, are applied to control the system. The amplitude and phase of each microwave pulses can be modulated to realize the CNOT gate. The CNOT gate is designed so that the state of qubit 2 is flipped iff qubit 1 is in state $|1\rangle$. To make the CNOT gate robust against the noises, we consider the quasi-static distributions of the noises in the optimization of the pulse sequence. The performance function is defined as the average gate fidelity

$$F'_{\text{a}} = \int d\delta_{0,1} \int d\delta_{1,1,\,\text{rel}} \int d\delta_{0,2} \int d\delta_{1,2,\,\text{rel}} P_{0,1}(\delta_{0,1}) P_{1,1,\,\text{rel}}(\delta_{1,1,\,\text{rel}}) P_{0,2}(\delta_{0,2}) P_{1,2,\,\text{rel}}(\delta_{1,2,\,\text{rel}}) F'_{\text{seq, err}}, \tag{44}$$

where $P_{0,1}(\delta_{0,1})$ and $P_{1,1,\,\text{rel}}(\delta_{1,1,\,\text{rel}})$ ($P_{0,2}(\delta_{0,2})$ and $P_{1,2,\,\text{rel}}(\delta_{1,2,\,\text{rel}})$) describe the distributions of quasi-static dephasing noise and control field fluctuation on NV 1 (NV 2). The distribution $P_{0,1}(\delta_{0,1})$ ($P_{0,2}(\delta_{0,2})$) is mainly due to the ^{13}C nuclear spin bath surrounding the NV 1 (NV 2). Since ^{13}C is naturally abundant, the distribution $P_0(\delta_0)$ in the main text is a typical estimation of $P_{0,1}(\delta_{0,1})$ ($P_{0,2}(\delta_{0,2})$). The distributions $P_{1,1,\,\text{rel}}(\delta_{1,1,\,\text{rel}})$ and $P_{1,2,\,\text{rel}}(\delta_{1,2,\,\text{rel}})$, mainly depending on the microwave generator, are consistent with $P_1(\delta_1)$ which can be obtained from experiment data. Considering the distributions, a pulse sequence for the CNOT gate can be optimized to achieve an average gate fidelity $F'_{\text{a}} = 0.9926$ by our method. The pulse sequence is shown in Supplementary Figure **11**. Thus our method can be applied to realize *robust* and *high fidelity* two-qubit gate on spatially separated NV centers.

Supplementary References

1. Nielsen, M. A. A simple formula for the average gate fidelity of a quantum dynamical operation. *Phys. Lett. A* **303**, 249-252 (2002). 14

2. Bowdrey, M. D., Oi, D. K.L., Short, A. J., Banaszek, K. & Jones, J. A. Fidelity of single qubit maps. *Phys. Lett. A* **294**, 258-260 (2002). 14

3. Khaneja, N., Reiss, T., Kehlet, C., Schulte-Herbrüggen, T. & Glaser, S. J. Optimal control of coupled spin dynamics: design of NMR pulse sequences by gradient ascent algorithms. *J. Magn. Reson* **172**, 296-305 (2005). 15, 18

4. Pedersen, L. H., Møller, N. M. & Mølmer, K. Fidelity of quantum operations. *Phys. Lett. A* **367**, 47-51 (2007). 15

5. Wang, X. *et al.* Composite pulses for robust universal control of singlet-triplet qubits. *Nature Commun.* **3**, 997 (2012). 16

6. Wimperis, S. Broadband, Narrowband, and Passband Composite Pulses for Use in Advanced NMR Experiments. *J. Magn. Reson. A* **109**, 221-231 (1994). 16

7. Bando, M., Ichikawa, T., Kondo, Y. & Nakahara, M. Concatenated composite pulses compensating simultaneous systematic errors. *J. Phys. Soc. Jpn.* **82**, 014004 (2013). 17

8. Kabytayev, C. *et al.* Robustness of composite pulses to time-dependent control noise. *Phys. Rev. A* **90**, 012316 (2014). 17

9. Epstein, R. J., Mendoza, F. M., Kato, Y. K. & Awschalom, D. D. Anisotropic interactions of a single spin and dark-spin spectroscopy in diamond. *Nature Phys.* **1**, 94-98 (2005). 19

10. Waldherr, G. *et al.* Quantum error correction in a solid-state hybrid spin register. *Nature* **506**, 204-207 (2014). 20

11. Moussa, O., da Silva, M. P., Ryan, C. A. & Laflamme, R. Practical experimental certification of computational quantum gates using a twirling procedure. *Phys. Rev. Lett.* **109**, 070504 (2012). 20

12. Rong, X. *et al.* Implementation of dynamically corrected gates on a single electron spin in diamond. *Phys. Rev. Lett.* **112**, 050503 (2014). 21

13. van der Sar, T. *et al.* Decoherence-protected quantum gates for a hybrid solid-state spin register. *Nature* **484**, 82-86 (2012). 21, 22

14. Knill, E. *et al.* Randomized benchmarking of quantum gates. *Phys. Rev. A* **77**, 012307 (2008). 23

15. Ryan, C. A., Laforest, M. & Laflamme, R. Randomized benchmarking of single- and multi-qubit control in liquid-state NMR quantum information processing. *New J. Phys.* **11**, 013034 (2009). 24

16. Brown, K. R. *et al.* Single-qubit-gate error below 10^{-4} in a trapped ion. *Phys. Rev. A* **84**, 030303 (2011). 24

17. Harty, T. P. *et al.* High-Fidelity Preparation, Gates, Memory, and Readout of a Trapped-Ion Quantum Bit. *Phys. Rev. Lett.* **113**, 220501 (2014). 24

18. Benhelm, J., Kirchmair, G., Roos, C. F. & Blatt, R. Towards fault-tolerant quantum computing with trapped ions. *Nature Phys.* **4**, 463-466 (2008). 24

19. Chow, J. M. *et al.* Universal quantum gate set approaching fault-tolerant thresholds with superconducting qubits. *Phys. Rev. Lett.* **109**, 060501 (2012). 25

20. Neumann, P. *et al.* Quantum register based on coupled electron spins in a room-temperature solid. *Nature Phys.* **6**, 249-253 (2010). 28

21. Grinolds, M. S. *et al.* Subnanometre resolution in three-dimensional magnetic resonance imaging of individual dark spins. *Nature Nanotech.* **9**, 279-284 (2014). 28
